# Benefits and Risks of the Hormetic Effects of Dietary Isothiocyanates on Cancer Prevention

**DOI:** 10.1371/journal.pone.0114764

**Published:** 2014-12-22

**Authors:** Yongping Bao, Wei Wang, Zhigang Zhou, Changhao Sun

**Affiliations:** 1 Norwich Medical School, University of East Anglia, Norwich, Norfolk, United Kingdom; 2 Department of Cardiovascular Medicine, Affiliated hospital of Nantong University, Nantong, Jiangsu, P. R. China; 3 School of Public Health, Harbin Medical University, Harbin, Heilongjiang, P. R. China; Southern Illinois University School of Medicine, United States of America

## Abstract

The isothiocyanate (ITC) sulforaphane (SFN) was shown at low levels (1–5 µM) to promote cell proliferation to 120–143% of the controls in a number of human cell lines, whilst at high levels (10–40 µM) it inhibited such cell proliferation. Similar dose responses were observed for cell migration, i.e. SFN at 2.5 µM increased cell migration in bladder cancer T24 cells to 128% whilst high levels inhibited cell migration. This hormetic action was also found in an angiogenesis assay where SFN at 2.5 µM promoted endothelial tube formation (118% of the control), whereas at 10–20 µM it caused significant inhibition. The precise mechanism by which SFN influences promotion of cell growth and migration is not known, but probably involves activation of autophagy since an autophagy inhibitor, 3-methyladenine, abolished the effect of SFN on cell migration. Moreover, low doses of SFN offered a protective effect against free-radical mediated cell death, an effect that was enhanced by co-treatment with selenium. These results suggest that SFN may either prevent or promote tumour cell growth depending on the dose and the nature of the target cells. In normal cells, the promotion of cell growth may be of benefit, but in transformed or cancer cells it may be an undesirable risk factor. In summary, ITCs have a biphasic effect on cell growth and migration. The benefits and risks of ITCs are not only determined by the doses, but are affected by interactions with Se and the measured endpoint.

## Introduction

The term ‘hormesis’ is often used by toxicologists to refer to a ‘biphasic dose response to an environmental agent characterized by low dose stimulation and by high dose inhibitory or toxic effect’ [1], [2]. The hormesis concept is the most fundamental dose-response relationship in the biomedical, nutrition and toxicological sciences [1]. In a comprehensive review, Calabrese provided evidence that more than a hundred anti-tumour agents enhanced the proliferation of human tumour cells at low doses in a manner fully consistent with the hormetic dose-response relationship [2]. One of the interesting characteristics of such dose-responses was that they occurred in most types of tumour cells and were independent of organ. Recent findings suggest that some phytochemicals exhibit biphasic dose responses in cells with low doses activating signalling pathways that result in increased expression of genes encoding cytoprotective proteins and antioxidant enzymes [3]. The dietary hormetic compounds identified so far include resveratrol, epigallocatechin gallate (EGCG), curcumin, quercetin, allicin, capsaicin, carnosic acid and sulforaphane (SFN) [4]–[8]. From an evolutionary perspective, the noxious properties of phytochemicals have an important protective role in dissuading insects and fungi from damaging plants. However, the relatively small doses of phytochemicals ingested by humans that consume these plants are not toxic and instead induce mild cellular stress responses. This phenomenon has been widely described as ‘hormesis’ or adaptive dose response in the fields of biology and medicine [4], [9], [10].

The isothiocyanate (ITC), SFN (4-methylsulfinylbutylisothiocyanate), was first isolated from the commonly-consumed cruciferous vegetable, broccoli and is one of the most potent naturally-occurring inducers of the Kelch-like ECH-associated protein 1 (Keap1)-nuclear factor erythroid 2-related factor 2 (Nrf2)-antioxidant response elements (ARE) pathway [11]. The induction of Nrf2 protects normal cells from free-radical mediated oxidative stress via upregulation of chemoprotective genes, and the action of SFN is based on its ability to induce a Nrf2-driven enzyme quinone reductase (NQO1) [12]. In the 20 years subsequent to its discovery, the protective effects of SFN have been demonstrated in various cell culture systems and animal models, with the result that SFN is by far the most extensively studied ITC from cruciferous vegetables. The anti-carcinogenic mechanisms of ITCs have also been well-documented, including up-regulation of phase II detoxification enzymes, anti-inflammation, promotion of cell cycle arrest and apoptosis [13]–[17]. During the last decade, Keap1-Nrf2-ARE has been considered as a critical anti-cancer pathway in chemoprevention [18]-[20]. However, more recently, there have been some deleterious reports of Nrf2, including promotion of tumour cell growth and chemoresistance [21]–[25]. In order to survive, cancer cells may hijack the Nrf2 pathway which upregulates a battery of antioxidant enzymes, thereby maintaining a favourable redox balance in order to acquire malignant properties [26]. Overexpression of Nrf2 could enhance cell proliferation and cause resistance to chemotherapeutic interventions in some types of cancer, including human lung and pancreatic cancers [27], [28]. A few previous investigations have shown that SFN exhibits a dose-dependent effects on cell proliferation in cultured tumour cell lines and normal cells including human mesenchymal stem cells [29]–[31]. In the present study, we showed that SFN exhibited a hormetic dose response on cell growth, migration and angiogenesis. Whether the hormetic effect is beneficial or harmful depends on the selected endpoint and/or the nature of the cells (normal or tumour). Although the term hormesis is employed by toxicologists to describe a bell-shaped dose response, characterized by a beneficial effect at low doses and a toxic (or inhibitory) activity at high doses, this expression of low dose benefit might not be true for the effect of ITCs in cancer chemoprevention. Since hormesis shows little selectivity, the biological effects of ITCs on normal cells and tumour cells will differ. From this perspective, a low dose effect of ITCs in promoting tumour cell proliferation and migration in animal models must be evaluated prudently. Thus, a precise strategy that aims to optimise the beneficial effects and minimise the risk of ITCs should be developed with care in relation to cancer prevention and treatment.

## Results

### Effects of ITCs on cell growth

**Figure 1:**
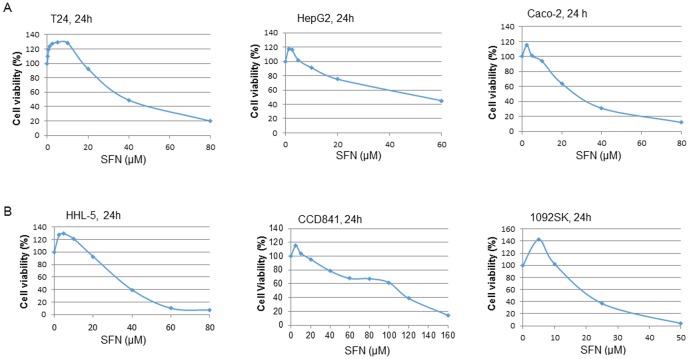
Effects of SFN on the proliferation of normal and tumour cells. When cells grew to 70–80% confluence, a range of doses of SFN (0–160 µM) were added to the cell culture medium for 24–48 h. The control cells were treated with DMSO (0.1%), and cell viability was determined by the MTT cell proliferation assay (CCD-1092SK cell viability was determined by WST-1 assay according to manufacturer's instructions [88]). Each data point represents the mean ± SD of at least 5 replicates. Statistical significance from the control, *p<0.05, or **p<0.01. A: results from bladder cancer T24, hepatoma HepG2, and colon cancer Caco-2 cells. B: Results from immortalised hepatocyte HHL-5, colon epithelial CCD841, and skin fibroblast CCD-1092SK cell lines.

Due to the nature of the hormetic dose response, there is no selectivity of ITCs on cell growth, so it is likely that ITCs can promote tumour cell growth at low doses. In several *in vitro* cell culture studies, low concentrations of SFN have been shown to promote tumour cell growth, but no detailed discussion or suggestions for follow-up studies to investigate the mechanisms were provided [32]–[34]. At low concentrations, ITCs have been shown to induce proliferation and/or protect cells against a toxic agent, H_2_O_2_, in Caco-2 cells [30] and in hepatocytes [29]. Fig. 1A shows the effects of SFN on cell growth, with lower doses (1–5 µM) promoting cell growth (20–43% greater than the control) and high doses (10–40 µM) inhibiting cell growth in a number of tumour cell lines, namely, bladder cancer T24, hepatoma HepG2, and colon cancer Caco-2. Similar dose response effects were found in normal cell lines including immortalised hepatocyte HHL-5, colon epithelial CCD841 and skin fibroblast CCD-1092SK cell lines (Fig. 1B).

### Effects of SFN on cell migration

**Figure 2:**
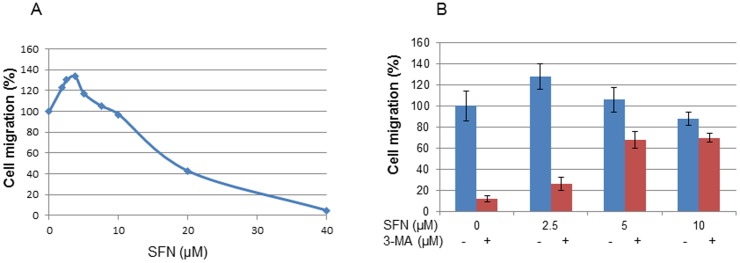
Effects of SFN and 3-MA on cell migration. A: After starvation overnight, bladder cancer T24 cells were treated with SFN at the concentrations indicated for 24 h, cell migration was measured by a cell migration assay using the ThinCert cell culture inserts (Greiner Bio-One Ltd.). Each bar represents the mean ± SD of 3 replicates. B: Effect of pre-treatment of 3-MA on cell migration. DMSO (0.1% was used as a control). Statistical significance from the control, *p<0.05, or **p<0.01.

Fig. 2A shows a bell-shaped dose response of SFN on bladder cancer T24 cell migration. SFN at 2.5 and 3.75 µM increased tumour cell migration to 128 and 133% in comparison with corresponding controls. Such SFN-induced cell migration is associated with the ability of SFN to activate autophagy. When an autophagy inhibitor, 3-methyladenine (3-MA), was used it alleviated SFN (2.5 µM)-induced cell migration from 128 to 26% although it has less inhibitory effect on SFN treatments at 5 or 10 µM (Fig. 2B). Moreover, 3-MA also decreased the migration of non-SFN treated cells to 12% of the control.

### ITCs and activation of Nrf2

**Figure 3:**
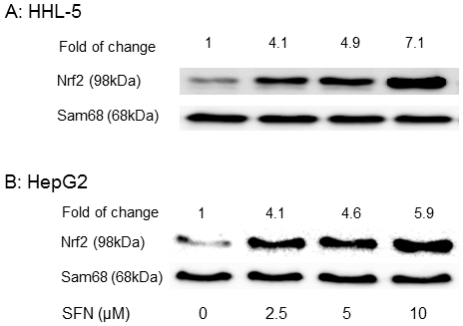
Effect of SFN on translocation of Nrf2 into cell nucleus. Nrf2 was detected in nuclear extracts from cells exposed to SFN (0, 2.5, 5 and 10 µM) for 24 h, using a Western blot assay. Control cells were treated with DMSO (0.1%). A: immortalised human hepatocyte HHL-5; B: human heptoma HepG2 cells.

SFN is an activator of Nrf2 via which it can up-regulate more than a hundred protective genes, including most antioxidant and chemopreventive enzymes [11], [35]. There is no doubt that up-regulation of Nrf2-ARE pathway is beneficial in normal cells, i.e. activation of Nrf2 and its driven cytoprotective enzymes can be protective against oxidative damage and it has been suggested that activation of the Nrf2 signalling pathway can thus be a promising strategy in cancer prevention [36]. But, ITCs have no selectivity towards either normal or tumour cells with regards to Nrf2 activation. Nrf2 can be hijacked by tumour cells [26], and a recent report suggests that Nrf2 is a protooncogene which modulates tumour cell growth [37]. In transformed cells, Nrf2 may promote cell growth or cause chemoresistance [38]. In this study, SFN (2.5–10 µM) induced similar levels of translocation of Nrf2 into the nucleus of normal human hepatocytes HHL-5 (4.1–7.1 fold), and hepatoma HepG2 (4.1–5.9 fold) cells (Fig. 3).

### Protective role of low dose ITC treatment against oxidative damage

**Figure 4:**
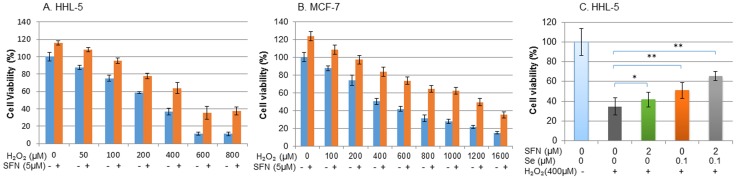
Effect of pre-treatment of cells with SFN protect against H_2_O_2_-induced cell death. Cells were cultured in 96 well plates. When they reached 70–80% confluence, cells were pre-treated with SFN (5 µM) for 24 h (HHL-5, A) or 48 h (MCF-7, B). The cell culture medium was replaced with H_2_O_2_ at the concentrations indicated for a further 24 h. C: HHL-5 cell were pre-treated with SFN (2 µM) and Se (0.1 µM) for 24 h before exposure to H_2_O_2_ (400 µM) for a further 24 h. The cell viability was measured using MTT assay. Statistical significance from corresponding controls: *p<0.05; **p<0.01.

In the fields of biology and medicine, hormesis is defined as an adaptive response of cells and organisms to a moderate stress. A mild stress induces the activation of signalling pathways such as Nrf2, NF-kB, Sirtuin, FOXO, hypoxia-inducible factor (HIF) thus leading to intrinsic changes (e.g. induction of antioxidant enzymes) that can confer resistance to more severe stress [4], [6]. Fig. 4A and 4B show that pretreatment of HHL-5 and MCF-7 cells with 5 µM SFN offered protection against H_2_O_2_-induced cell death, i.e. cell viability increased from 36.6 to 63.9%; and from 50.3 to 83.7% with 400 µM H_2_O_2_ treatments, respectively. Moreover, the protective effect of pretreatment with SFN (2 µM) on H_2_O_2_-induced cell death could be enhanced by cotreatment with selenium (Se) in HHL-5 cells (Fig. 4C), i.e. H_2_O_2_ decreased cell viability to 34.8% in HHL-5 cells but when cells were pre-treated with SFN (2 µM), or Se (0.1 µM) for 24 h, the cell viability increased to 41.7 and 51%, respectively and co-treatment SFN and Se increased cell viability to 65.5%. This protective effect may be involved in either chemoprotection or chemoresistance, depending on the nature of the cells.

### Biphasic effects of SFN on angiogenesis

**Figure 5:**
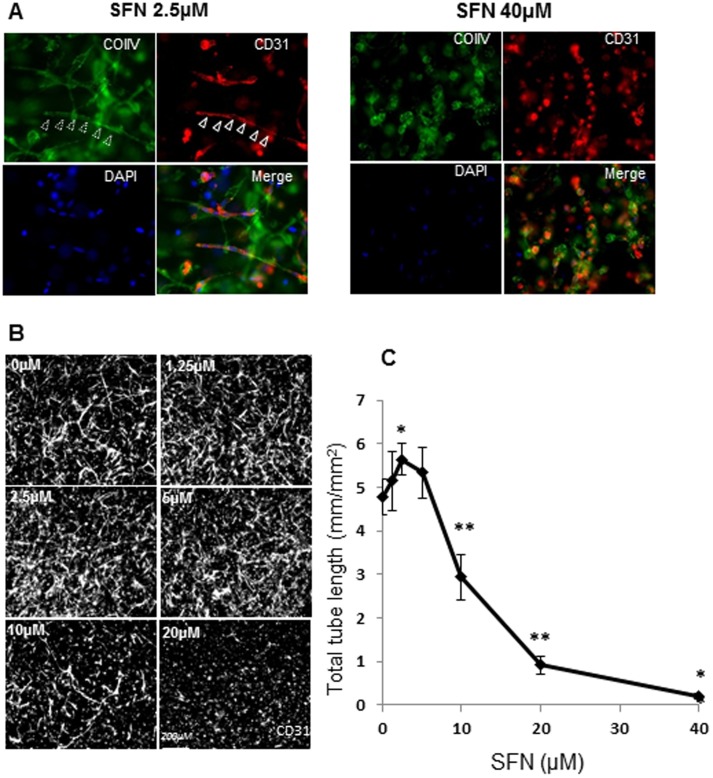
Effect of SFN on endothelial tube formation in a 3-D angiogenesis assay. Culture medium supplemented with SFN (0–40 µM) was added to the top of 3-D collagen gels and then changed every 24 h with fresh SFN added. 3-D gels were fixed at day 5, immunostained with CD31 (red) and collagen type IV (green), and counterstained with DAPI (blue). (A): Low magnification pictures were taken from five random fields of each sample and calculated for average tube length. (B) Representative pictures are shown in triple staining with higher magnification. Data are expressed as mean ± SD (n = 5) (C). *P<0.05; ** P<0.01 compared to untreated control.

Angiogenesis (new blood vessel growth) is crucial in the development and spread of a variety of human cancers. It is, therefore, important to examine the anti-angiogenic effects of potential anti-cancer agents. In contrast, inadequate blood supply to the heart and other tissues, resulting from insufficient new blood vessel growth, is a feature of many cardiovascular diseases. SFN has been shown to inhibit angiogenesis at high concentrations [39]. In this study, SFN at 2.5 µM promoted tube formation to 118% of the control, i.e. total tube length was 4.78 mm/mm^2^ in control and 5.65 mm/mm^2^ in SFN (2.5 µM) treated cells (Fig. 5). SFN at 5 µM showed a less significant promotion (111% relative to the control), whereas 10 and 20 µM SFN inhibited tube formation significantly (decreased to 61 and 20% of the control, respectively). SFN at low dose promoted the formation of a continuous basement membrane around endothelial tubes; whereas at high doses of SFN, fragmented basement membranes were found (Fig. 5A). These data suggest that for anti-angiogenesis a relatively high dose of SFN should be used since a lower dose may promote angiogenesis. However, the stimulating effect of low doses on new blood vessel formation could be beneficial in patients with cardiovascular diseases.

## Discussion

### Hormetic effect of ITCs on cell growth, migration and angiogenesis

The hormetic zone concentrations (approximately 1–5 µM) of ITCs that are added in cell culture could readily be achieved in human plasma after consumption of a meal rich in cruciferous vegetables, or from extracts or supplements [40]–[44]. Table 1 shows the plasma levels of ITCs measured in several human studies (see also reference [45]). SFN is derived from the action of the endogenous enzyme, myrosinase on the glucosinolate, glucoraphanin which is found in cruciferous vegetables. The glucosinolate contents of common Brassica are available from a database developed by McNaughton and Marks [46]. The highest glucosinolate value was from cress (389 mg/100 g fresh weight) while the lowest value was from Chinese cabbage (20 mg/100 g fresh weight), although cultivar type and growing conditions both influence these figures. Broccoli contains 61.7 mg/100 g (19.3–127.5 mg glucoraphinin/100 g) [46], which is equivalent to 141.3 µmol SFN/100 g (44.2–292.1 µmol/100 g fresh weight) if the conversion is 100% efficient. Food processing and cooking conditions are crucial factors in influencing the activity of myrosinase, and subsequent formation of ITCs [47]. The main influence on the ensuing production of ITCs *in vivo* is how the brassica vegetables have been cooked [48]. Extensive studies of SFN have provided convincing evidence that SFN is a chemopreventive agent [49], [50]; and the mechanisms of its action involves the induction of phase II enzymes, cell cycle arrest and apoptosis [16], [51].

**Table 1:** Human studies with plasma levels of dietary ITCs.

Study type	Subjects (n)	Dose	Plasma conc. (µM)	Refs
Metabolisms, pharmacokinetics	4	200 µmol ITCs (largely SFN)	0.94–2.27	Ye *et al*., 2002 [40].
Metabolism	16	GST(+): 107 & 345.8 µmol SFN; GST(-): 95 & 342 µmol SFN	2.2; 7.3 2.3; 7.4	Gasper *et al*., 2005 [41].
Metabolism	4	70 or 120 µmol SFN	0.9 or 2.1	Cramer *et al*., 2011 [42].
Bioavailability	12	150 µmol glucoraphanin	2.2	Clarke *et al*., 2011 [43].
Pharmacokinetics	4	100 g watercress	0.928 (±0.25)	Ji *et al*., 2003 [44].

In general, findings from epidemiological studies on the association between vegetable intake and cancer risk are inconsistent. A high intake of cruciferous vegetables has, however, been shown to decrease the risk of several types of cancer, including those of colon and lung [52], [53]. If the hormetic effects of ITCs are involved in cancer growth, the overall biological impact of cruciferous vegetable on cancer risk becomes much more complicated. However, if a low dose of ITCs promotes cancer cell growth it may help to explain why epidemiological studies do not show a consistent association between cruciferous vegetable intake and the risk of cancer. Therefore, it is crucial to understand the mechanisms of action of the hormetic effects of ITCs. In *in vitro* cell cultures, the mechanisms by which low doses of SFN promote cell growth may be related to the effect SFN has on the activation of growth promoting molecules (such as HER2, RAS, RAF, MEK, ERK, PI3K, AKT and mTOR), signal transduction pathways such as NF-kB, FOXO, HIF, Nrf2, autophagy and receptors [54]–[56].

Autophagy involves the formation of double-membraned vesicles (autophagosomes), which encapsulate the cytoplasm and organelles and fuse with lysosomes, leading to degradation of the contents of the vesicle [57]. SFN is known to be an inducer of autophagy [58], but it is unclear how induction of autophagy is associated with suppression of cell migration. Other potential targets of SFN may include matrix metalloproteinases (MMPs), microtubules, collagens and integrins, survivin and zinc finger E-box binding homeobox 1 (ZEB1) [59]. A very recent study suggests that activation of autophagy is associated with chemoresistance, and that histone deacetylase (HDAC)10 protects neuroblastoma cells from cytotoxic agents by mediating autophagy [55]. This work indicates that co-treatment with HDAC10 inhibitor and a chemotherapeutic drug (doxorubicin) is a promising way to improve treatment response. Another study suggests that Notch activation is largely dispensable for SFN-mediated inhibition of cell migration in human prostate cancers [60], and this could be a therapeutic advantage as Notch activation is common in human prostate cancers. High constitutive levels of Nrf2 occur in many tumours, whilst overexpression of Nrf2 in cancer cells protects them from the cytotoxic effects of anticancer therapies, resulting in chemoresistance [22], [61]. There are interactions between ITCs and Se in the up-regulation of thioredoxin reductase (TR-1) and glutathione peroxidase 2 (GPx2) [30] and it is clear that ITCs and Se exhibit a plethora of multi-targeted effects in cancer chemoprevention. Interestingly, Se also promotes the migration and invasion of prostate cancer PC3 cells [62].

### Assessment of the hormetic effect of ITCs

**Figure 6:**
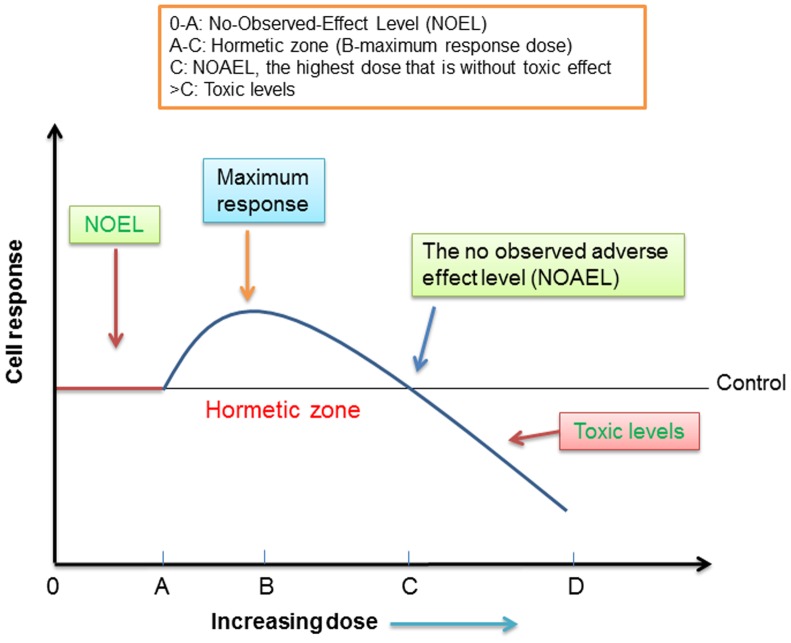
A schematic diagram on the hormetic effect of ITCs. For all cell types, dosage range 0-A is safe. In the majority of diets, the intakes of hormetic phytochemicals are likely to fall within this safe range. For normal cells, dose B could be used promote new blood vessel formation or promote wound healing; doses >C are toxic. For tumour cells, doses between A and C should be avoided; and doses >C to D could be used for chemotherapy.

Consumption of cruciferous vegetables would not only provide ITCs but also contribute other nutrients and phytochemicals, including tocopherols, flavonoids, ascorbate and Se. These components could counteract/interact with the prooxidant/antioxidant activities of ITCs. Based on the hormetic nature of ITCs, consumption of a quantity of cruciferous vegetables that provide a hormetic level of ITCs in plasma could be a risk factor for those who have transformed cells in the body. A schematic diagram for analysing the benefits and risks of dietary ITCs is proposed in Fig. 6. For all dietary compounds and toxic substances, “the dose makes the poison” [wording simplified from “all things are poison, and nothing is without poison; only the dose permits something not to be poisonous” (Paracelsus, 1493–1541)]. For dietary ITCs, there should be a “no effect level” prior to the detection of any biological effects. Indeed, the level of ITCs in the plasma of a majority of the population is likely to be much lower than sub-µM and may not exert any biological effects on cells. However, following increased intakes, such as in the trials listed in Table 1 or for individuals taking supplements, the plasma ITC levels could reach the hormetic zone concentrations. Typical characteristics of the hormetic zone (dose A-C) includes low concentrations stimulating and high concentrations inhibiting effects. For SFN, the hormetic zone is found to be 1–5 µM in the *in vitro* cell culture experiments, although the dose-effects found *in vitro* experiments should not be directly extrapolated to humans. It is possible that the hormetic zone and the No Observed Adverse Effect Level (NOAEL, dose C) in humans is significantly different. In order to maximise the beneficial effect and minimise the risk, both genetic factors and interactions between dietary components should be considered. For example, genetic polymorphisms of glutathione transferases (GSTs) affect SFN metabolism and the risk of cancer [63]. On the other hand, supplementation with cruciferous vegetables increased GSTA1/2 activity, the effect being most marked in GSTM1-null/GSTT1-null men [64]. Although there are currently few epidemiological studies that employ genotyping, research of this nature will increase in the future and it is likely the nutrigenetics will provide a basis for personalised medicine and nutrition. Interactions between bioactive phytochemicals and nutrients may contribute to the overall benefits and risks of ITCs depending on the health status of the individuals. The inductions of Nrf2 and antioxidant enzymes such as TR-1 could also be of either benefit or risk depending on the nature of the target cells (normal *vs* tumour).

### Where are we now? How can we maximise the benefits and minimise the risks?

Thirty years ago, researchers focused on the potential toxic (goitrogenic) properties of glucosinolate breakdown products [65]. In 1992, sulforaphane was isolated from broccoli and anti-carcinogenic studies were based on its potent activity in the induction of phase II enzymes [12], [66]. Over the last decade, many Nrf2 inducers including ITCs, resveratrol, catechin, cucurmin, and quercetin have been reported [67], [68] with both chemopreventive and oncogenic activities [69]–[71]. Recently, two Nrf2 inhibitors, brusatol (from the seeds of *Brucea sumatrana*) and trigonelline (from coffee) were reported to enhance the efficacy of anticancer therapy [72], [73]. Moreover, Nrf2 knockdown has been shown to inhibit tumour growth, increase the efficacy of chemotherapy in cervical cancer [74], and inhibit the angiogenesis of rat cardiac micro-vascular endothelial cells under hypoxic conditions [75]. Therefore, it is clear that the role of Nrf2 in cancer development is a topic of controversy and Nrf2 activators such as SFN and other ITCs may contribute both benefits and risks in cancer development.

An understanding of the complex plethora and divergent natures of ITCs and other dietary Nrf2 activators and their hormetic dose responses, combined with an accurate diagnosis (stage of cancer), and genetic analysis may, in the not-too-distant future, initiate the significant potential that personalised medicine may have. New diagnostic techniques exploiting gold nanoparticles can spot tumour-like masses as small as 5 mm in the liver [76]. Gold nanoparticles with a polyelectrolyte coating can make even smaller tumours visible through X-ray scatter imaging, thereby enabling earlier diagnosis. Once tumours can be diagnosed at such a very early stage, a potential therapeutic approach could be the nanoencapsulation of cancer-fighting phytochemicals or drugs through monitored and targeted delivery [77]. But, it must be remembered that ITCs at high concentrations are also toxic towards normal cells. Adverse effects have been reported in *in vitro* studies using 10–30 µM SFN, including induction of DNA, RNA and mitochondrial damage [78]–[80]. Moreover, there was also a case report of liver toxicity in an individual who consumed 800 ml broccoli soup a day for 4 weeks [81]. Low levels of ITCs can generate reactive oxygen species (ROS), and activate Nrf2-ARE to switch on antioxidant enzymes. Although high levels of ROS can damage protein, lipids and DNA in cells, low levels of ROS can play an important role in immune defence, antibacterial action, vascular tone, and signal transduction [82]. Recently, James Watson hypothesised that diabetes, dementias, cardiovascular disease and some cancers are all linked to a failure to generate sufficient ROS [83]. The challenge is to define the balance between the generation of ROS and the antioxidant capacity in each type of cells. For dietary ITCs, it is important to define the optimal range of intakes for promoting health. Nevertheless, further human studies are required to establish the personalised optimal doses, safety and efficacy profiles using more sensitive biomarkers.

## Materials and Methods

### Materials

Sulforaphane was purchased from Enzo Life Sciences (UK). Sodium selenite, dimethylsulfoxide (DMSO), hydrogen peroxide, Bradford reagent, methylthiazolyldiphenyl-tetrazolium bromide (MTT), phenylmethylsulfonyl fluoride (PMSF), and all other materials and reagents were purchased from Sigma-Aldrich (UK). Rabbit polyclonal primary antibodies to Nrf2, Sam68 and horseradish peroxidase (HRP)-conjugated goat anti-rabbit IgG as secondary antibodies were all obtained from Santa Cruz Biotechnology Inc. (Heidelberg, Germany). Anti-collagen IV and anti-human CD31/PECAM-1 were purchased from Millipore and BD Biosciences (UK), respectively. Secondary antibodies conjugated with Cy2 and Cy3 were purchased from Jackson Immuno Research (UK). Mini-complete proteinase inhibitor and WST-1 reagent were purchased from Roche Applied Sciences (UK). Electrophoresis and Western blotting supplies were supplied by Bio-Rad (UK). The enhanced chemiluminescence (ECL) kit was purchased from GE Healthcare (UK).

### Cell culture

Immortalised human hepatocytes (defined as HHL-5) were kindly supplied by Dr Arvind Patel, Medical Research Council (MRC) Virology Unit (Glasgow, UK) [84]. All other cell lines were purchased from ATCC. Cells were routinely cultured in DMEM supplemented with foetal bovine serum (10%), 2 mM glutamine, penicillin (100 U/ml) and streptomycin (100 µg/ml) under 5% CO_2_ in air at 37°C.

### Cell proliferation assay

The cell proliferation MTT assay was employed to detect the toxicity of SFN (1–160 µM) on cultured cells. When cells were at approximately 70–80% confluence, cells were exposed to various concentrations of SFN for different times using DMSO (0.1%) as control. After all treatments, the medium was removed, 5 mg/ml MTT was added, and incubated at 37°C for 1 h to allow the MTT to be metabolized. Then the formazan produced was re-suspended in 100 µl DMSO per well. The final absorbance in the wells was recorded using a microplate reader (BMG Labtech Ltd, UK) at a wavelength of 550 nm and a reference wavelength of 650 nm.

### Cell migration assay

Cell migration was quantified using a ThinCert cell culture inserts cell migration assay (Greiner Bio-One Ltd.). After overnight starvation in serum free medium, cells were treated with various concentrations of SFN for 24 h, the cells migrating through a PET membrane were labelled fluorescently with Calcein-AM and quantified by microplate reader (BMG Labtech Ltd, UK) with an excitation wavelength of 485 nm and emission wavelength of 525 nm.

### Protein extraction and Western blot Analysis

For total protein, HHL-5 cells were washed twice with ice-cold PBS, harvested by scraping in 20 mM Tris-HCl (pH 8), 150 mM NaCl, 2 mM EDTA, 10% glycerol, 1% Nonidet P40 (NP-40) containing mini-complete proteinase inhibitor. The cell suspensions were placed in an ice bath for 20 min and then centrifuged at 12,000 g for 15 min at 4°C. Supernatant was collected and the protein concentration determined by the Bradford Brilliant Blue G dye-binding assay of using BSA as a standard. For the nuclear protein, the extraction was performed by using a Nuclear Extract Kit (Active Motif, UK), following the manufacturer's instructions.

Protein extracts were heated at 95°C for 5 min in loading buffer and loaded onto 10% SDS-polyacrylamide gels together with a molecular weight marker. After routine electrophoresis and transfer, the polyvinylidene difluoride (PVDF) membrane was blocked with 5% fat free milk in PBST (0.05% Tween 20) for 1 h and incubated with a specific primary antibody in 5% milk in PBST for 1 h. The membrane was washed three times for 45 min with PBST and then incubated with the secondary antibody diluted with 5% milk in PBST for 1 h. After three further washes for 45 min with PBST, the antibody binding was determined using an ECL kit (GE Healthcare, UK) and densitometry was measured by Fluor Chem Imager (Alpha Innotech, San Leandro, CA).

### Angiogenesis assay - tube formation in a 3-D model

Human umbilical vein endothelial cells (HUVEC) and pericytes (PVC) were co-cultured in collagen type I gel as described previously [85]. SFN (0–40 µM) was added to the medium (top of 3-D collagen gel) and the medium was changed every 24 h with fresh SFN added. At day 5, samples were fixed, immunostained with CD31 and collagen type IV and counterstained with DAPI. Magnification pictures were taken from five random fields of each sample and average tube length measured.

### Statistics

Data are represented as the mean ± SD. The differences between the groups were examined using one-way ANOVA test, or student's t-test. A *p* value <0.05 was considered to be statistically significant. IC_50_ values of SFN and H_2_O_2_ were determined using CalcuSyn Software (Biosoft, UK).

## Conclusions and Future Perspectives

Based on findings from the research reported here, greater effort should be expended on the evaluation of the interactive/synergistic effects on the cancer risk of various phytochemicals and phytochemical-rich foods. Risk/benefit assessment of ITCs and other dietary bioactives may be linked to genotype, health status or tumour stage, and of course the dose, all of which must be included in future research priorities. More precise dietary guidelines and policies for cancer prevention could also be developed based on the understanding of these fundamental factors. There are at least five ongoing human trials using SFN or broccoli sprout preparations registered with http://www.clinicaltrials.gov/ and it will be of great interest to study the results in coming years. In the absence of precise knowledge in these areas, it is considered prudent to study the molecular mechanisms of the interactions between ITCs and other bioactives/nutrients in cell cultures and animal models prior to undertaking large, very expensive human trials. In this sense, β-carotene has been a good example. In observational studies, high intake of carotenoids from food has been associated with reduced risk of cancer. However, observational studies are inherently unreliable and it would be a big mistake to conduct human trials without having sufficient information about the mechanisms of action in cells. In intervention trials, β-carotene supplements have not been found to offer any benefits; in fact, when taken in high doses for a long period of time, they slightly increased the risk of some forms of cancer [86]. However, this is an area of activity that is rapidly developing and this assessment may well need to be revisited in the light of emerging scientific data. These results show that low concentrations of ITCs especially SFN may be potentially beneficial or harmful, depending on the endpoint of interest and the cell type, i.e. beneficial to normal angiogenesis and harmful in promotion of cancer cell growth. SFN is important because it is present in our normal diet from cruciferous vegetables and also because of its commercial applications (there are many different brands of broccoli extracts marketed as supplements). Based on the hormetic dose response, nutraceutical producers should carefully consider the efficacy of the application of ITC/SFN-rich products/supplements. On the basis of their biphasic effects on cell growth and migration, there is no doubt that ITCs belong to the so-called hormetic class of phytochemicals.

In summary, low concentrations of ITCs can potentially be either beneficial or harmful. Since there is little selectivity in the hormetic effect, the benefits or risks of ITCs at lower doses could be different in normal and tumour cells. In tumour cells, low doses of SFN could have the capability to increase the risk of tumour development. In contrast, in normal endothelial cells, SFN could be significantly cardio-protective (angiogenetic). This type of conflict between beneficial and harmful effects is not uncommon and may be related to the different biological systems, tissues and chemical agents under investigation [87]. The evidence regarding the hormetic dose response induced by SFN is obvious, but the relevant molecular mechanisms are not fully understood, and thus deserve greater attention in future research. Nutrition scientists and oncologists should be aware of the potential risks of dietary ITCs, especially of the possible role of hormesis if they are used as food supplements. Finally, the majority of the available evidence described above is based on *in vitro* cell culture experiments. Research is also needed to evaluate the relative risks, as well as benefits, of the hormetic effects in medium- to long-term supplementation with dietary ITCs and other phytochemicals in animal studies and small scale human trials.
